# Mediation Effect of Suicide-Related Social Media Use Behaviors on the Association Between Suicidal Ideation and Suicide Attempt: Cross-Sectional Questionnaire Study

**DOI:** 10.2196/14940

**Published:** 2020-04-28

**Authors:** Xingyun Liu, Jiasheng Huang, Nancy Xiaonan Yu, Qing Li, Tingshao Zhu

**Affiliations:** 1 Institute of Psychology Chinese Academy of Sciences Beijing China; 2 Department of Psychology University of Chinese Academy of Sciences Beiijng China; 3 Department of Social and Behavioural Sciences City University of Hong Kong Hong Kong China (Hong Kong); 4 Department of Computing The Hong Kong Polytechnic University Hong Kong China (Hong Kong)

**Keywords:** suicidal ideation, suicide, attempted, social media, suicide-related social media use behaviors

## Abstract

**Background:**

A limited number of studies have examined the differences in suicide-related social media use behaviors between suicide ideators and suicide attempters or have sought to elucidate how these social media usage behaviors contributed to the transition from suicidal ideation to suicide attempt.

**Objective:**

Suicide attempts can be acquired through suicide-related social media use behaviors. This study aimed to propose 3 suicide-related social media use behaviors (ie, attending to suicide information, commenting on or reposting suicide information, or talking about suicide) based on social cognitive theory, which proposes that successive processes governing behavior transition include attentional, retention, production, and motivational processes.

**Methods:**

We aimed to examine the mediating role of suicide-related social media use behaviors in Chinese social media users with suicidal risks. A sample of 569 Chinese social media users with suicidal ideation completed measures on suicidal ideation, suicide attempt, and suicide-related social media use behaviors.

**Results:**

The results demonstrated that suicide attempters showed a significantly higher level of suicidal ideation (*t*_563.64_=5.04; *P*<.001; two-tailed) and more suicide-related social media use behaviors, which included attending to suicide information (*t*_567_=1.94; *P*=.05; two-tailed), commenting on or reposting suicide information (*t*_567_=2.12; *P*=.03; two-tailed), or talking about suicide (*t*_542.22_=5.12; *P*<.001; two-tailed). Suicidal ideation also affected suicide attempts through the mediational chains.

**Conclusions:**

Our findings thus support the social cognitive theory, and there are implications for population-based suicide prevention that can be achieved by identifying behavioral signals.

## Introduction

### Background

Suicide has been a critical public health problem, with approximately 1 million people committing suicide worldwide annually [1]. Although significant efforts have been made in the field of suicide prevention, suicide rates have seen an increase in recent decades [2]. In fact, it was reported that one-third of people with suicidal ideation would take actions to kill themselves [3]. Although a large amount of evidence has been reported in relation to risk factors (eg, depression, alcohol use disorders, and hopelessness) that can be associated with suicidal ideation, little is known regarding how suicide ideators become suicide attempters [4]. As such, to facilitate precise suicide risk assessment and prevention, it is crucial to identify the mechanism that illustrates how suicide ideators progress into suicide attempters.

The ideation-to-action framework states that the development of suicidal ideation and the progression from ideation to suicide attempt are two distinct processes [5]. Pain and hopelessness account for suicidal ideation, whereas suicide capabilities transform suicidal ideation into suicide attempt. Suicide capabilities include acquired (eg, habituation to fear and pain involved in death), dispositional (eg, genetic fearlessness and pain tolerance), and practical (eg, access to means and knowledge of attempted lethality) capabilities that may serve in attempting suicide. Indeed, existing research about the progression from ideation to suicide attempt has mainly focused on disorders such as posttraumatic stress disorder, depression disorder, and anxiety disorder [4]. To elucidate how suicide capabilities can turn suicidal ideation into suicide attempt, studies investigating observable behaviors as warning signals that foreshadow how suicidal ideation turns into suicide attempt are warranted, and such information would enhance population-based suicide screening and improve the efficiency of suicide prevention [6].

Today, the internet can provide new data sources because people record their lives in varying degrees on the website, and it has become an active interaction platform for young people, where they sometimes exchange thoughts portraying a susceptibility to suicidality [7]. Live streams of suicides are found on social media [8], and people often use such media to share their distress [7]. Indeed, young people are reluctant to disclose their suicidal signs in medical visits, but they are somehow willing to share these on social media platforms such as Facebook and Twitter [9]. This aforementioned research indicates the possibility of using web-based behaviors in social media as helpful markers for suicide risk assessment. Although web suicidal behaviors may be a proxy for suicidal ideation, the web behavioral pattern of suicide attempters is not clear [10]. Researchers found that factors such as high levels of internet use, internet addiction, and exposure to websites with self-harm or suicidal content were particularly associated with suicidal behavior [11]. However, we are still unclear as to how web social media use behaviors contribute to the progression of suicidal ideation to suicide attempt [10,12].

People like to create and exchange user-generated content on social media [13]. In Sina Weibo (similar to Twitter), people can post microblogs (ie, tweets); can search, read, comment on, and repost others’ microblogs; and would also be able to join in the groups they are interested in [14]. O'Connor and Kirtley found that exposure to suicide or suicidal behavior can turn suicide ideators into suicide attempters [15]. As such, social media platforms can function like a hatchery for suicidal behaviors in certain ways. Indeed, social cognitive theory suggests that people can acquire new behaviors through observing others. The theory proposes that successive processes governing observational learning include attentional, retention, production, and motivational processes [16]. Thus, suicidal behaviors can be seen as modeled behaviors, which are acquired through observing others’ suicidal behaviors on social media. Consequently, the social cognitive theory has been used to examine the effect of mass media on suicide [16,17]. Fu et al [17] found a positive association between media influences and suicidal ideation based on social cognitive theory. However, they only took the internet as a traditional form of media, akin to television, and neglected the interactions that people made on the website. Therefore, we extended the existing study by using the social cognitive theory in this analysis to illustrate the effects of suicide-related social media use behaviors on the process from suicidal ideation to suicide attempt. On the basis of previous research [14], we mainly focused on 3 suicide-related social media use behaviors: (1) attending to suicide information (*attending to*), (2) commenting on or reposting suicide information (*commenting-reposting*), or (3) talking about suicide (*talking about*).

Attentional process is the first step of observational learning. The attentional process revolves around what people selectively pay attention to about the observed models and what message they procure from a wealth of information. Preconceptions, value preferences, and other factors determine this process [16]. People with suicidal ideation would have preconceptions of suicide, and they may inadvertently come across or actively seek suicidal posts on the website because social networking sites are important sources of suicide stories [18]. It has been confirmed that suicidal ideation was found to be significantly associated with the accessing of suicide or self-injury information on the website [19]. The reciprocal relationship between suicidal ideation and suicide-related information is thus an upward spiral feedback loop [17], particularly when the personalized recommender system works. Suicidal posts often include evocative words and images, which have been proven to be a risk factor for suicide behaviors [20]. Considerable evidence has indicated that exposure to stories of others’ suicides can influence young people at suicide risk to attempt the same act [17,18]. Therefore, *attending to* is the first suicide-related social media use behavior we suggested that would be related to suicide attempts.

The processes for observational learning are retention and production. Retention involves the process of remembering the modeled activities. This process would involve not only simply copying but also proactively reconstructing the observed events. Production would be the deep processing of retention when learned behavior is generated through a “conception-matching process” (ie, through a production process, cognitive activities are developed into corresponding explicit behaviors) [16]. These two processes are closely linked. As we focus on warning behaviors in this study, we joined these two processes together. When compared against traditional media, social networking sites offer additional opportunities for transmission of suicide portrayals and knowledge of suicide among friends [18], which means that the internet offers a large number of opportunities to retain and produce suicide information the users can attend to through functions such as commenting or reposting. Through commenting or reposting, people with suicidal ideation may process suicide information more deeply than they would by just attending to suicide information. The suicide news would become part of their memory, and they would put themselves into the suicide stories they saw, imagining how the stories could happen to them. This process may then accelerate the progression from suicidal ideation to suicide attempt. Adolescents use public websites to display comments about their attitude toward these suicide stories or their own suicidal ideation or suicide plan, actions that have been confirmed to have a close relation to suicide attempts [15]. Therefore, *commenting-reposting* is the second suicide-related social media use behavior we suggested that would be related to suicide attempts.

Finally, the motivational process is the final stage to make the decision on whether one would act out on the acquired modeled behaviors. Social cognitive theory differentiates internal acquisition from external behaviors because people would not show all the things that they have ever learned. They would have a higher possibility of performing the modeled behaviors that they felt were similar to their situation, and they value the outcomes [16]. By attending to suicide-related information on social media, suicidal social media users may adore what the models did to end a miserable life by committing suicide, particularly when this practice is shared by similar people. They may participate in online suicide groups to discuss with like-minded individuals about successful suicide cases, general issues associated with suicide, or their own suicide plan [21,22]. Moreover, they could directly talk about their own substantial suicide plan on the website publicly. Those explicit expressions of suicidal behaviors can not only send out signals that the subjects have a strong mind for suicide but also obtain feedback from others, who may in turn encourage them to commit suicide because the interaction can diminish the fear about death through further mental imagery or the normalizing, reinforcing, and even glorification of suicide [23]. In particular, the interactions may foster peer pressure to die by suicide or facilitate suicide pacts [22]. Such pacts are not rare globally in today’s society because they improve the possibility of successful death by suicide [24-26]. It has been demonstrated that talking about suicidal ideation on Twitter can be significantly associated with having a suicide plan and attempting suicide [20,27]. Therefore, *talking about* is the final suicide-related social media use behavior we proposed that would be related to suicide attempt.

### Objectives

In summary, the mechanism of the progression from suicide ideators to suicide attempters among social media users is unclear. On the basis of earlier research [14], we focused on 3 suicide-related social media use behaviors in sequence (ie, *attending to*, *commenting-reposting*, and *talking about*) according to social cognitive theory [16], as behavioral warning signals that may serve to illuminate the transmitting path.

There are 2 hypotheses for suicidal social media users:

H1: Compared with suicide ideators, suicide attempters would report a higher level of suicide-related social media use behaviors, including *attending to*, *commenting-reposting*, and *talking about.*

H2: Suicidal ideation would predict suicide attempt through the mediating chains of *attending to*, *commenting-reposting*, and *talking about*.

## Methods

### Participants

As the most popular social media platform in China, Sina Weibo has nearly 300 million users, and most of them are younger than 30 years [28,29], which makes it a perfect platform to prevent Chinese youth from committing suicide. We found a blogger on Weibo who committed suicide because of depression, and many people follow her suicide note even though she died 7 years ago. Until October 1, 2019, over 1 million comments were posted on her web suicide note. This unique post has become a “secret garden” to attract many people who suffer from depression or suicidal ideation. We manually annotated the comments for this post from March 2016 to June 2016, and 4616 social media users were identified as suicidal. The reason why we chose those comments is that it is nearly impossible to search suicidal posts randomly from Weibo, as, on average, 100 million new posts are generated every day on this platform [28]. We took this web suicide note as a breakthrough to trace suicidal social media users.

We sent a direct message that provided social support, referrals, and a link to questionnaires to these 4616 suicidal users. More details can be found in the study by Liu et al [30]. We reported the results according to the Checklist for Reporting Results of Internet E-Surveys [31]. A total of 725 individuals completed the questionnaires after signing informed consent, with no compensation being provided. Ethical approval was obtained from the Institute of Psychology, Chinese Academy of Sciences.

### Measures

The demographic information of participants was collected, including their sex, age, education level, marital status, and living status (living alone, with family or partner, with friends, or with others).

Suicidal ideation was measured by the 4-item version of the Adult Suicidal Ideation Questionnaire (ASIQ) [32]. Each item is rated on a 7-point scale rating from 0 (I never have this thought) to 6 (almost everyday life) to measure the severity of suicidal ideation. A sample item was “I thought about killing myself.” We used the total average score of the 4 items to represent suicidal ideation as a continuous variable. Both the original [32] and Chinese version [33] of the scale displayed good psychometric properties. The internal consistency, indicating whether items measure a unidimensional concept and whether a value higher than 0.70 is desirable [34], in this study was high (alpha=.91).

Suicide attempt was measured by one item “Have you ever tried to kill yourself?” Participants responded with binary choices (yes or no). As suicide attempt is a binary dependent variable, participants responded no (0) or yes (1) for suicide attempt. Participants who responded no to the suicide attempt item were grouped as suicide ideators, and those who responded yes to the suicide attempt item were categorized as suicide attempters [35].

Suicide-related social media use behaviors were developed by our team. This measure consists of *attending to* (2 items: “Attending to suicide news” and “Attending to friends who said they wanted to commit suicide”), *commenting-reposting* (2 items: “Commenting on or reposting suicide news” and “Commenting on or reposting other people’s posts about killing themselves”), and *talking about* (2 items: “Talking about suicide in online suicide communities” and “Talking on the website about one's own concrete plan to commit suicide”). Participants indicated how often it had occurred in the last year based on a 5-point Likert scale that ranged from 1 (never) to 5 (mostly). The internal consistency values of the 3 subscales were 0.64, 0.74, and 0.72, respectively, in this study. The measurement items can be found in Multimedia Appendix 1.

### Data Analysis

**Figure 1:**
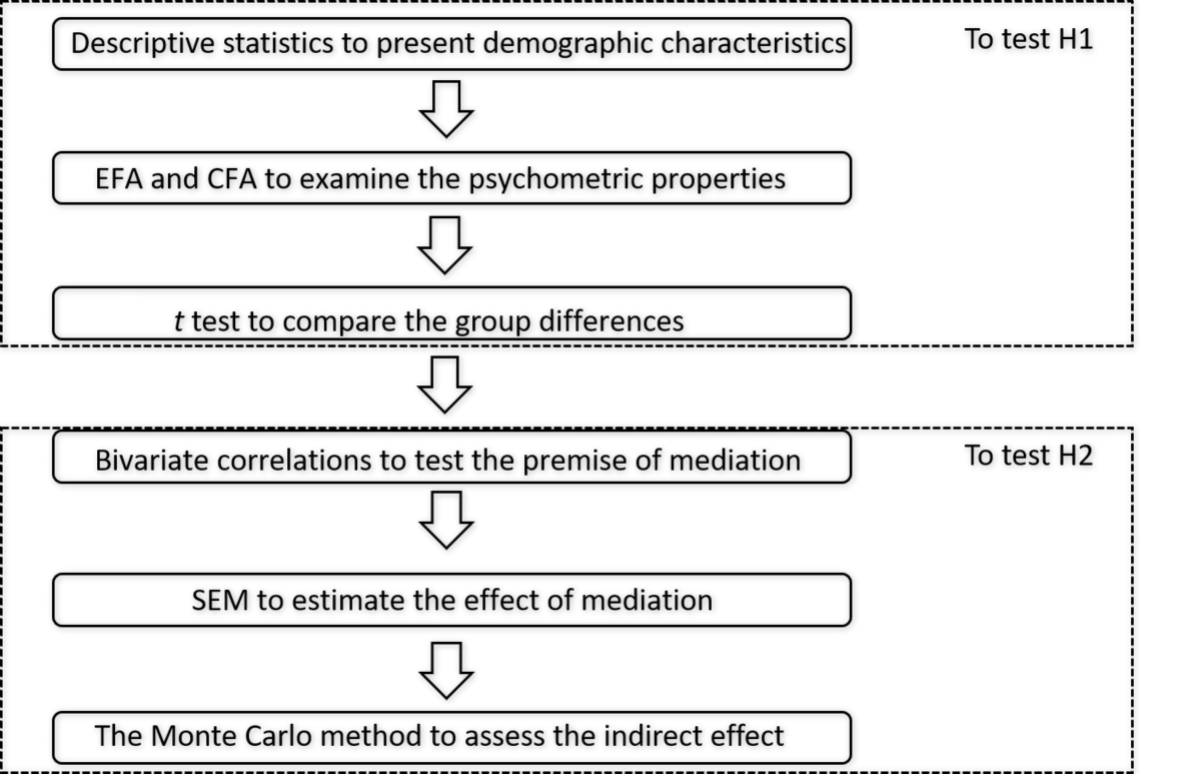
The flowchart for data analyses. CFA: confirmatory factor analysis; EFA: exploratory factor analysis; SEM: structural equation modeling; H1: hypothesis 1; H2: hypothesis 2.

Mplus7 (Muthen and Muthen, Beijing, China) and SPSS21 (SPSS Inc, Beijing, China) were used for the statistical analysis. Descriptive statistics for demographic characteristics and study variables were also tabulated. Exploratory factor analysis (EFA) and confirmatory factor analysis (CFA) were conducted to examine the psychometric properties of the self-developed measure of suicide-related social media use behaviors. *t* test was used to compare the differences between suicide ideators and suicide attempters. In addition, bivariate correlations were assessed. To evaluate the mediation model, structural equation modeling (SEM) was used to estimate latent variables because the latent variables can decrease measurement error and consider measurement and equation modeling at the same time [36]. Model fit was evaluated using several fit indices: (1) the chi-square (χ^2^) test of model fit, (2) the root mean square error of approximation (RMSEA), (3) the comparative fit index (CFI), (4) the Tucker Lewis Index (TLI), and (5) the weighted root mean square (WRMR) because of the presence of binary data; that is, participants received 0 or 1 for suicide attempt. An RMSEA value of less than 0.06, a CFI at or above 0.90, a TLI at 0.90 or higher, and a WRMR value less than 1.00 indicate a relatively good fit [37,38]. Finally, the Monte Carlo method was used to further test the indirect effect in the mediation. Rather than testing new models with different constellations of the listed variables, the Monte Carlo method showed the estimated sizes of different indirect effects in the original model, so the model fit statistics were the same as those in the original model [39] (Figure 1).

## Results

### Descriptive Statistics Results

After excluding respondents that reported conflicting or missing data (146/569, 20.1%) and no suicidal ideation (the ASIQ score=0; 10/569, 1.4%), there were 569 valid participants who reported a certain degree of suicidal ideation. This number exceeds the required sample size to test the study question, as the minimum sample size is 295 (power=0.80; alpha=.05; H0: RMSEA=0, H1: RMSEA=0.05; and df=36) [40]. The demographic characteristics of participants are displayed in Table 1. We grouped the participants into suicide ideators and suicide attempters according to their reports as to whether they had previously attempted suicide. The mean ages for all participants, suicide ideators, and suicide attempters were 21.94 (SD 3.31) years, 22.07 (SD 3.59) years, and 21.82 (SD 3.02) years, respectively. The age difference between the 2 groups was not significant (*t*_540.19_=0.90, *P*=.37, two-tailed). As shown, there was no difference in demographic variables between the 2 groups. Most participants were unmarried young females with college degrees, and most of them were living with others (friends or families).

**Table 1:** Demographic characteristics of participants by suicidal status (N=569).

Characteristic		Total (N=569), n (%)	Suicide ideators (n=277), n (%)	Suicide attempters (n=292), n (%)		Chi-square value (*df*)		*P* value
**Sex**					**0.25 (1)**		**.62**	
	Male	78 (13.7)	40 (14.4)	38 (13.0)				
	Female	491 (86.3)	237 (85.6)	254 (87.0)				
**Educational level**					**2.46 (2)**		**.29**	
	Primary	28 (4.9)	11 (4.0)	17 (5.8)				
	Secondary	97 (17.1)	53 (19.1)	44 (15.1)				
	Tertiary	444 (78.0)	213 (76.9)	231 (79.1)				
**Marital status**					**1.43 (2)**		**.49**	
	Single	531 (93.3)	256 (92.4)	275 (94.2)				
	Married	24 (4.2)	12 (4.3)	12 (4.1)				
	Divorced or others	14 (2.5)	9 (3.3)	5 (1.7)				
**Living status**					**4.95 (4)**		**.29**	
	With families	201 (35.3)	90 (32.5)	111 (38.0)				
	With partner	33 (5.8)	16 (5.8)	17 (5.8)				
	With friends	231 (40.6)	115 (41.5)	116 (39.7)				
	Alone	75 (13.2)	44 (15.9)	31 (10.6)				
	Others	29 (5.1)	12 (4.3)	17 (5.8)				

### Results to Test Hypothesis 1

To examine its psychometric properties, we split the sample (n=569) into two random and equal halves. We randomly used half of the sample to conduct EFA and the other half to conduct CFA. See Table 2 for the results of the EFA; the factor number was set to 3. The 3 factors, in total, explained 77.4% of the total variance. The construct validity was supported by the EFA results.

The CFA model showed a good fit to the data: χ^2^_6_=2.1 (*P*=.09), RMSEA=0.06, CFI=0.99, TLI=0.97, and SRMR=0.02. Both the EFA and CFA results indicated that the scale had good psychometric properties.

On the basis of whether the participants attempted suicide or not, they were grouped as suicide ideators or suicide attempters as mentioned above. The group differences on study variables by suicide status are shown in Table 3. The attempters group showed a significantly higher level of suicidal ideation (*t*_563.64_=5.04 *P*<.001; two-tailed) than that shown by the ideators group. Consistent with hypothesis 1, the attempters reported more *attending to* (*t*_567_=1.94; *P*=.05; two-tailed), *commenting-reposting* (*t*_567_=2.12; *P*=.03; two-tailed), and *talking about* (*t*_542.22_=5.12; *P*<.001; two-tailed) behaviors than those reported by suicide ideators.

Descriptive statistics and correlations of study variables are shown in Table 4. As shown, suicidal ideation (*r*=0.21; *P*<.001) was significantly correlated with suicide attempt. The 3 suicide-related social media use behaviors (ie, *attending to:* *r*=0.08, *P*=.05; *commenting-reposting:* *r*=0.09, *P*=.03; and *talking about:* *r*=0.21, *P*<.001) were significantly correlated with suicide attempt. Therefore, we conducted subsequent mediation analysis with path analysis.

**Table 2:** Exploratory factor analysis of 6-item suicide-related social media use behaviors.

Items	Attending to^a^	Commenting-reposting^b^	Talking about^c^
Attending to suicide news	0.88	N/A^d^	N/A
Attending to friends who said they wanted to commit suicide	0.74	N/A	N/A
Commenting on or reposting suicide news	^—e^	0.71	N/A
Commenting on or reposting other people’s posts about killing themselves	N/A	0.90	N/A
Talking about suicide in online suicide communities	N/A	N/A	0.85
Talking on the website about one’s own concrete plan to commit suicide	N/A	N/A	0.82

^a^Attending to suicide information.

^b^Commenting on or reposting suicide information.

^c^Talking about suicide.

^d^N/A: not applicable.

^e^Loading <0.04 was not shown in the table.

**Table 3:** Group differences on study variables by suicidal status (N=569).

Variables	Suicide ideators (n=277), mean (SD)	Suicide attempters (n=292), mean (SD)	*t* test (*df*)	*P* value	Effect size
Suicidal ideation	2.34 (1.69)	3.10 (1.92)	−5.04 (563.64)	<.001	0.42
Attending to^a^	2.86 (0.77)	3.00 (0.91)	−1.94 (567)	.05	0.17
Commenting-reposting^b^	2.41 (0.95)	2.59 (1.04)	−2.12^e^ (567)	.03	0.18
Talking about^c^	1.70 (0.88)	2.14 (1.15)	−5.12^a^ (542.22)	<.001	0.43

^a^Attending to suicide information.

^b^Commenting on or reposting suicide information.

^c^Talking about suicide.

**Table 4:** Descriptive statistics and bivariate correlations of the study variables.

Variables		Correlation coefficient (*r*)					Value, mean (SD)
		Suicidal ideation	Suicide attempt	Attending to^a^	Commenting-reposting^b^		
**Suicidal ideation**						**2.73 (1.85)**	
	*r*	N/A^c^	N/A	N/A	N/A		
	*P* value	N/A	N/A	N/A	N/A		
**Suicide attempt**						**292 (51.3)**	
	*r*	0.21	N/A	N/A	N/A		
	*P* value	<.001					
**Attending to**						**2.93 (0.85)**	
	*R*	0.31	0.08	N/A	N/A		
	*P* value	<.001	.05				
**Commenting-reposting**						**2.50 (1.00)**	
	*R*	0.30	0.09	0.52	N/A		
	*P* value	<.001	.03	<.001			
**Talking about^d^**						**4.73 (1.42)**	
	*R*	0.46	0.21	0.46	0.56		
	*P* value	<.001	<.001	<.001	<.001		

^a^Attending to suicide information.

^b^Commenting on or reposting suicide information.

^c^N/A: not applicable.

^d^Talking about suicide.

### Results to Test Hypothesis 2

**Figure 2:**
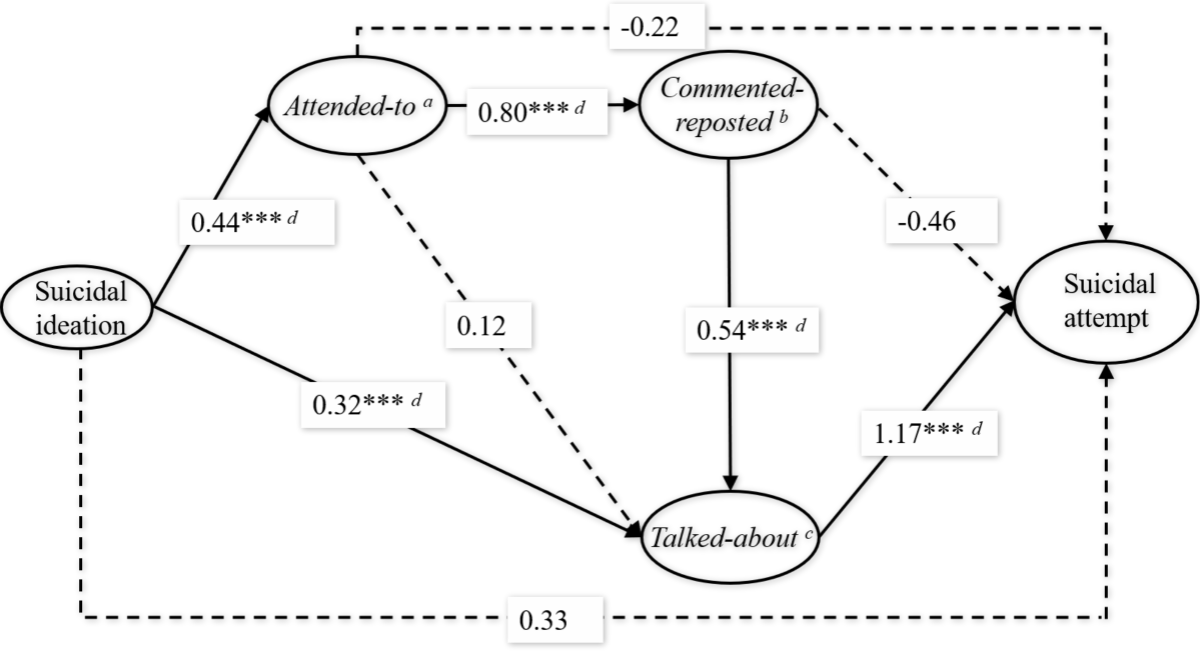
The mediating effect of social media use behaviors in the association between suicidal ideation and suicide attempt. a) Attended to suicide information; b) Commented on or reposted suicide information; c) Talked about suicide; d) *P*<.001.

Results of SEM are depicted in Figure 2. Model indices were acceptable, with χ^2^_36_=2.1 (*P*<.001), RMSEA=0.05, CFI=0.94, TLI=0.90, and WRMR=0.48. When considering the *attending to*, *commenting reposting*, and *talking about* variables, the association between suicidal ideation and suicide attempt was found to be no longer significant (β=.33; *P*=.14). Suicidal ideation was associated with a higher level of *attending to* (β=.44; *P*<.001) and *talking about* (β=.32; *P*<.001) behaviors. Only the *talking about* behavior (β=1.17; *P*<.001) was associated with suicide attempt. The relation between *attending to* and *talking about* behaviors was mediated by the *commenting-reposting* behavior. These associations supported hypothesis 2, which states that suicidal ideation predicted suicide attempts through the mediating chains of *attending to*, *commenting-reposting*, and *talking about* behaviors.

Further mediation analysis with the Monte Carlo method found significant indirect effects of (1) *attending to*, *commenting-reposting*, and *talking about* behaviors as the mediators between suicidal ideation and suicide attempt and (2) *attending to* and *commenting-reposting* behaviors as the mediators between suicidal ideation and the *talking about* behavior. In addition, *attending to* and *commenting-reposting* behaviors did not show significant mediation effects in the association between suicidal ideation and suicide attempt. The estimated mediation effects are shown in Table 5, which provide support for hypothesis 2.

**Table 5:** Estimated mediation effects of attending to, commenting-reposting, and talking about behaviors in the association between suicidal ideation and suicide attempt.

Mediation effect	Indirect effect	95% CI	*P* value
Suicidal ideation→*Attending to*^a^→*Commenting-reposting*^b^→*Talking about*^c^	0.19	0.11 to 0.27	<.001
Suicidal ideation→*Attending to*^a^→Suicide attempt	−0.10	−0.50 to 0.31	.64
Suicidal ideation→*Attending to*^a^→*Commenting-reposting*^b^→Suicide attempt	−0.16	−0.48 to 0.16	.32
Suicidal ideation→*Attending to*^a^→*Commenting-reposting*^b^→*Talking about*^c^→Suicide attempt	0.22	0.09 to 0.36	.001

^a^Attending to suicide information.

^b^Commenting on or reposting suicide information.

^c^Talking about suicide.

## Discussion

### Principal Findings

On the basis of the social cognitive theory, this study aimed to identify web-based behavioral markers in social media that could distinguish suicide ideators from suicide attempters and illuminate the transition mechanisms that led from suicidal ideation to suicide attempt. Our findings demonstrated that when compared with Chinese social media users who were suicide ideators, suicide attempters reported more suicide-related social media use behaviors. More importantly, the effect of suicidal ideation on suicide attempt was mediated by suicide-related social media use behaviors. Our findings, therefore, make a theoretical contribution to the field by providing behavioral markers in the progression from suicidal ideation to suicide attempt with the Chinese social media user population that has suicidal ideation. To our knowledge, this is the first study to focus on the identification of suicide-related social media use behaviors and illustration of the progression from suicidal ideation to suicide attempt.

Consistent with previous studies [4], no difference in sex, marital status, or educational level between suicide ideators and suicide attempters among Chinese social media users was found. Our findings also indicated that demographic characteristics are not helpful in distinguishing suicide ideators from suicide attempters in Chinese social media users, and there would be a need to use behavioral or other markers to differentiate between these 2 groups [6]. Interestingly, suicide attempters demonstrated more suicide-related social media active use behaviors than suicide ideators. Specifically, although they showed a marginal significance in the *attending to* behavior, they did report significantly more *commenting-reposting* and *talking about* behaviors as compared with suicide ideators. Our findings are also consistent with a Japanese study, which demonstrated that talking about one’s own suicidal ideation on Twitter was positively associated with suicide attempt [27]. Moreover, previous evidence has indicated that social media may exacerbate suicidal behaviors because social media users could receive descriptions of how to kill oneself in pro-suicide groups [22]. Owing to the fact that the behavioral patterns among social media users that contribute to the transition from suicidal ideation to suicide attempt are unclear, more research is required to investigate how these 2 groups are different in terms of *attending to*, *commenting-reposting*, *talking about,* and other suicide-related social media use behaviors (eg, logging in to suicide-related blogs or clicking “like” for suicidal posts). Our findings indicate that behavioral risks of suicide attempters will pave the way for the identification of high-risk cases based on the web-based surveillance of social media use behaviors. For example, a few useful programs have been using a similar approach to prevent suicide online, such as “Psymap” in China [41] and Facebook’s suicide-prevention tools [42]. These programs are helpful in detecting suicidal social media users through their suicide-related social media use behaviors, such as commenting on suicide notes. These existing programs have demonstrated feasibility and effectiveness in identifying suicidal cases with an attempt at monitoring their social media use behaviors. Our findings also provide empirical evidence as a rationale behind conducting these preventive measures. That being said, our study calls for more big-data studies on behavioral patterns of social media use among suicide attempters with interdisciplinary research involving psychology, psychiatry, computer science, and linguistics.

Interestingly, our findings demonstrated that suicidal ideation predicted suicide attempt through the mediating chains of social media use behaviors, which included the *attending to*, *commenting-reposting*, and *talking about* behaviors. Our results indicate that suicide-related social media use behaviors are a social learning process, which turns suicide ideators into suicide attempters through the process of attention (what people selectively notice, eg, attending to suicide information), retention, and production (what people constructively remember and what people externally acquire, eg, commenting on or reposting these posts) before finally moving to motivation (what people perform into action, eg, talking about their own suicide plan), as depicted in the social cognitive theory. Furthermore, our study demonstrated that the associations of *attending to* and *commenting-reposting* behaviors with suicide attempt were mediated by the *talking about* behavior. *Talking about* behavior made the biggest contribution in the mediating role in predicting suicide attempt. This result indicates that an individual may have a higher chance of committing suicide when he or she directly talks about suicide on the website compared with those who attend to suicide news and simply comment on or repost suicide information. This finding supports the social cognitive theory, which states that the attentional process, as well as the retention and production processes, contribute to behavioral acquisition, whereas the motivational process determines behavioral performance. From the attentional process to the motivational process, more processing and input are involved [16]. As the motivational process, talking about suicide, which requires an initiative with explicit expression, may involve more input and deeper processing than the *attending to* behavior, which is implicit, and the *commenting-reposting* behavior, which is responsive, do [17]. Our finding is consistent with previous results, which state that talking about suicide on the website is one of the most important warning signals for suicide [43]. Moreover, researchers have pointed out that suicide is only possible insofar as it is meaningful [44]. By talking about suicide on the website, suicidal people may complete the process of meaning-making, which leads to a higher possibility of suicide attempt. It is also worth noting that the association between suicidal ideation and the *talking about* behavior is significant. One possible explanation is that by being different from traditional media, such as newspaper and television [17], the internet provides a unique platform for direct interaction, thereby accelerating the social cognitive process to skip the beginning components (eg, attention, retention, and production) and reach the terminal component (ie, motivation).

Our mediation findings also provide insight into explaining how suicide capacity is acquired in social interaction online, potentially complementing the ideation-to-action framework [4]. This theory states that suicide capability is acquired through a habituation to fear and pain involved in death, access to means of committing suicide, knowledge of attempting deadliness, and so on [5]. Reading, commenting on, and reposting suicidal posts on the website, which often include evocative words and images [20], may also enhance one’s suicide capability by eliminating the fear of death and increasing the knowledge of how to die. The “upward spiral” feedback loop between suicide-related information and suicidal ideation [17] would be boosted in the online interaction, and suicide capability is, therefore, enhanced in a web-based context. Moreover, talking about one’s own suicidal ideation in social media may aggravate the situation because it may attract similar minds to attempt suicide together or normalize, reinforce, and even glorify suicide [23]. Hence, suicide capability may efficiently turn into action. Our study expands the ideation-to-action framework to a web-based situation by providing empirical evidence to confirm that *attending to*, *commenting-reposting*, and *talking about* behaviors are alarm signals that indicate the suicide ideators’ intent to commit suicide.

### Limitations

Although the study has made several contributions to the existing research, there are several limitations to this work. First, participants in this study were mainly suicidal, unmarried young females with college degrees. Our results are consistent with previous studies showing that single young females with a higher education were more inclined to talk about their suicidal ideation and seek help [30,45]. At the same time, because all those participants were recruited from one single social media blogging site, there is a possibility that they may differ from other suicidal social media users who do not comment on this post. For example, they may have a higher level of suicidal ideation or use Weibo more frequently. In addition, they may be affected by other users who also left comments on this post while they interact through comments [46]. Caution should be taken in generalizing our findings to a larger sample size in other blogging sites, social media platforms, and social media users with different demographic characteristics and suicidal status. Moreover, although the suicide-related social media use behaviors examined in this study (ie, *attending to*, *commenting-reposting*, and *talking about*) seem to occur widely, these results need to be replicated in different cultures. Second, a previous study reported that there are potential risks of online suicide intervention programs because of the possibility of increasing contagion among the participants [47]. In addition, the frequency of social media use was positively associated with suicide risk [46]. We only examined 3 suicide-related social media use behaviors in this study. Future research should consider investigating other suicide-related social media use behaviors, such as logging in to suicide blogs, following suicide-provoking or suicide-preventing public accounts, and clicking “like” for suicidal posts. Third, even though Weibo is popular in China, there are still some drawbacks; for example, the accuracy of a web-based suicide claim needs to be further confirmed [48]. Chinese people even use various metaphors to describe death [49]. These phenomena make identification more difficult. In addition, not like Facebook or Twitter, in which the users are globally distributed and use different languages, Weibo bloggers are mainly Chinese and most use Chinese. Moreover, Sina Weibo’s message limit to 140 characters may impede expression. Future study may pay attention to these language limitations to understand their impacts. We also suggest a combination of online logs and offline data such as school performance or health profile to identify suicide. Fourth, because of the cross-sectional study design, the associations presented in this study are correlational in nature. The effect of suicide-related social media use behaviors found in this study would need to be examined with longitudinal designs over various time frames (minutes, hours, days, weeks, and even months) [50]. Finally, this work relied on self-report data of suicidal issues, and this method is subject to potential biases. Future studies can consider adopting more objective indicators such as public log data because social media use behaviors can be recorded and observed from one’s account history. Of course, ethics, real-world implementation, and legal liability are complex issues that cannot be overlooked [51].

### Conclusions

This study aimed to investigate the behavioral markers available for distinguishing suicide ideators from suicide attempters and elucidate the behavioral process that transits from suicidal ideation to suicide attempt. Our findings demonstrated that suicide attempters showed a significantly higher level of suicidal ideation and more suicide-related social media use behaviors, including *attending to*, *commenting-reposting*, and *talking about* behaviors. Moreover, *attending to*, *commenting-reposting*, and *talking about* behaviors are chain mediators that convert suicidal ideation into suicide attempt. *Talking about* behavior mediates the relation between suicidal ideation and suicide attempt. These findings support the social cognitive theory and expand the ideation-to-action framework by clarifying the pathways of how suicide ideators turn into suicide attempters via the mediation role of suicide-related social media use behaviors. This study, therefore, paves the way for future research to focus on more behavioral markers that can serve in the identification of suicide attempters, subsequently contributing to the development of efficient and effective population-based suicide prevention.

## Appendix

Multimedia Appendix 1 The measurement items for the whole study.

## Abbreviations

ASIQ: Adult Suicidal Ideation Questionnaire
CFA: confirmatory factor analysis
CFI: comparative fit index
EFA: exploratory factor analysis
RMSEA: root mean square error of approximation
SEM: structural equation modeling
TLI: Tucker Lewis Index
WRMR: weighted root mean square
