# Supplementary material for: Mediation Effect of Suicide-Related Social Media Use Behaviors on the Association Between Suicidal Ideation and Suicide Attempt: Cross-Sectional Questionnaire Study
Source: J Med Internet Res. 2020 Apr 28;22(4):e14940. doi: 10.2196/14940 (PMC7218592; doi:10.2196/14940)
Supplement: Multimedia Appendix 1 [file jmir_v22i4e14940_app1.docx]

| Factors | Measure | Items |
| --- | --- | --- |
| Suicide ideation | The Adult Suicidal Ideation Questionnaire | I thought about killing myself |
|  |  | Others will be happier if I gone. |
|  |  | Suicide would solve my problems. |
|  |  | I would kill myself if I had chance. |
| Suicide attempt | One-item measure | Have you ever tried to kill yourself? |
| *Attended-to* ^a^ | Self-developed measure | Attended to suicidal news |
|  |  | Attended to friends who said they wanted to commit suicide |
| *Commented-reposted* ^b^ |  | Commented on / reposted suicide news |
|  |  | Commented on/reposted other people’s posts about killing themselves |
| *Talked-about* ^c^ |  | Talked about suicide in online suicidal communities |
|  |  | Talked on the website about one’s own concrete plan to commit suicide |

*^a^ Attended to:* Attended to suicidal information;

*^b^* *Commented-reposted*: Commented on/reposted suicidal information;

*^c^* *Talked-about:* Talked-about suicide;
